# Nanoimaging of Facet-Dependent Adsorption, Diffusion, and Reactivity of Surface Ligands on Au Nanocrystals

**DOI:** 10.1021/acs.nanolett.3c00250

**Published:** 2023-06-16

**Authors:** Lihi Rikanati, Hadar Shema, Tzipora Ben-Tzvi, Elad Gross

**Affiliations:** Institute of Chemistry and The Center for Nanoscience and Nanotechnology, The Hebrew University, Jerusalem 91904, Israel

**Keywords:** surface ligands, nanospectroscopy, nanocrystals, single particle

## Abstract

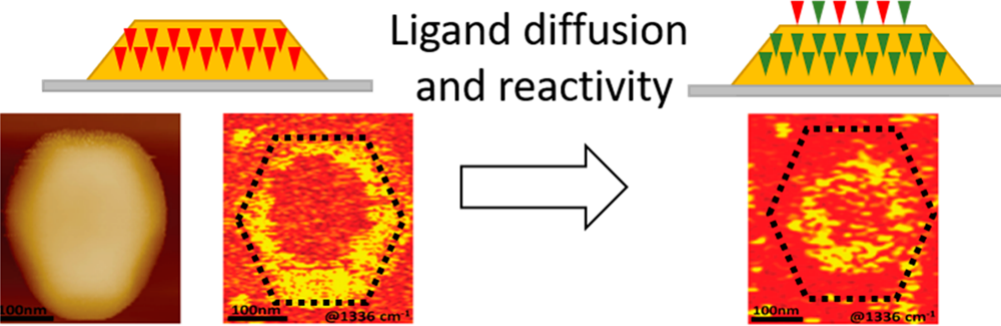

Analysis of the influence of dissimilar facets on the adsorption, stability, mobility, and reactivity of surface ligands is essential for designing ligand-coated nanocrystals with optimal functionality. Herein, para-nitrothiophenol and nitronaphthalene were chemisorbed and physisorbed, respectively, on Au nanocrystals, and the influence of different facets within a single Au nanocrystal on ligands properties were identified by IR nanospectroscopy measurements. Preferred adsorption was probed on (001) facets for both ligands, with a lower density on (111) facets. Exposure to reducing conditions led to nitro reduction and diffusion of both ligands toward the top (111) facet. Nitrothiophenol was characterized with a diffusivity higher than that of nitronaphthalene. Moreover, the strong thiol–Au interaction led to the diffusion of Au atoms and the formation of thiol-coated Au nanoparticles on the silicon surface. It is identified that the adsorption and reactivity of surface ligands were mainly influenced by the atomic properties of each facet, while diffusion was controlled by ligand–metal interactions.

Surface-ligands have been functioning as key parameters in directing the properties of metallic and semiconductor nanocrystals.^1,2^ Chemical modification of surface sites by ligands was utilized for regulating the solubility and availability of active components during synthesis,^3−6^ minimization of surface energy,^7,8^ enabling self-assembly,^9,10^ as well as encoding designated functionality.^11−21^ The properties of ligand-coated nanocrystals depend on the distribution, stability, and chemical reactivity of surface-ligands.^22−25^

Nanocrystals are constructed of several facets that vary in their atomic order, and these differences will modify ligands’ properties on each atomic facet.^26−29^ Therefore, analysis of the influence of various facets on the adsorption, stability, mobility, and chemical reactivity of ligands is essential for designing ligand-coated nanocrystals with specific functionality.

Conventional techniques such as X-ray photoelectron spectroscopy (XPS) and vibrational spectroscopy offer macroscopic analyses of surface-ligand properties.^30,31^ However, they cannot directly probe the facet-dependent ligand properties on nanocrystals. Conversely, while scanning tunneling microscopy (STM) offers high spatial resolution mapping of ligands orientation in highly ordered domains,^32−35^ it provides limited information about the chemical functionality and reactivity of ligands and their distribution in disordered domains.

High spatial resolution vibrational nanospectroscopy measurements can address this knowledge gap and provide unique insights into the distribution, stability and chemical reactivity of surface ligands on various domains.^36−42^ IR nanospectroscopy measurements already identified nanoscale disorder and inhomogeneity in the distribution of ligands on Au films^43^ and Au nanoparticles.^44^ The influence of nanoscale variations in surface structure and composition on the chemical reactivity of surface ligands was identified as well by IR and Raman nanospectroscopy measurements.^45−100^ However, a direct analysis, at the single-nanocrystal level, of the influence of different atomic facets on ligand properties has not yet demonstrated.

Au nanocrystals were prepared on Si(110) wafers with a size range of 100–600 nm (Figure S1). To identify the influence of various atomic facets on the adsorption and reactivity of chemically functionalized ligands, we have focused our study on analyzing the properties of ligands that were adsorbed on nanocrystals with a well-defined Wulff-like structure (Figure 1A,B).^58,59^ A lamella was extracted from the sample by focused ion beam (FIB), and transmission electron microscopy (TEM) images of the probed nanocrystal, along with analysis of the electron diffraction pattern (Figure S2), enabled the classification of its atomic facets (Figure 1C). This analysis validated that nanocrystals are constructed of a Wulff structure with a top (111) facet and alternating (111) and (001) side facets (Figure 1B).

**Figure 1:**
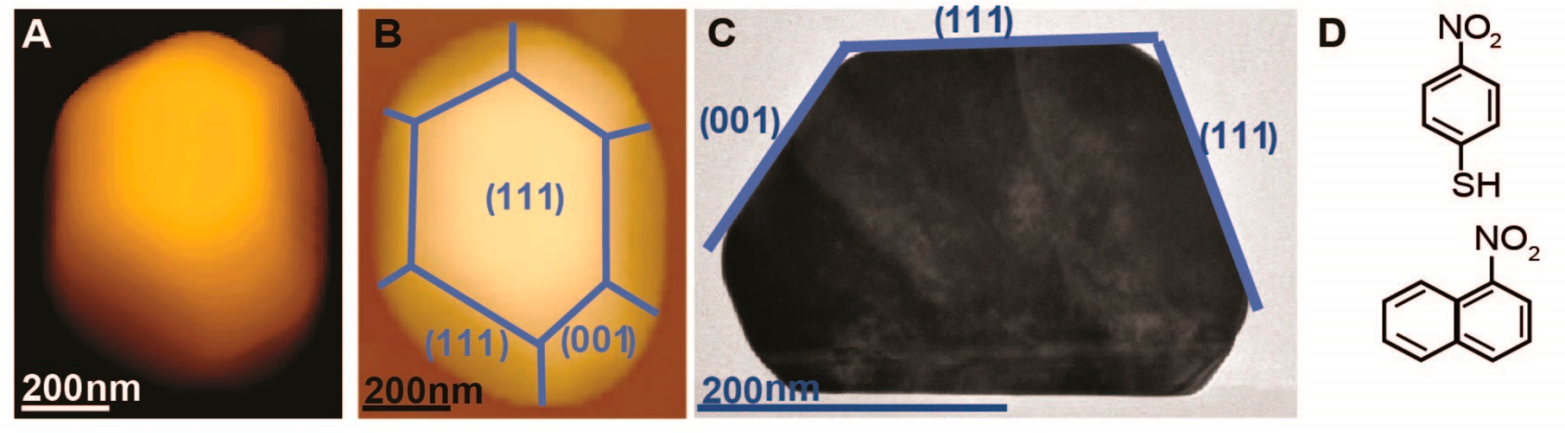
(A) AFM image of Au nanocrystal. (B) AFM image of nanocrystal shown in (A) with facet indexing based on TEM imaging (C) of a cross section of the Au nanocrystal. (D) Molecular structure of p-NTP and nitronaphthalene (top and bottom, respectively), which were used as surface ligands.

Para-nitrothiophenol (p-NTP) and nitronaphthalene (Figure 1D) were self-assembled on Au nanocrystals, and their vibrational signature was measured (Figure S3). Both p-NTP and nitronaphthalene are functionalized with a chemically active nitro group but differ in their surface interactions, while p-NTP is chemisorbed on the Au surface,^31,60^ nitronaphthalene forms van der Waals (VdW) interactions with the underlying Au atoms.^61^

High spatial resolution IR mapping of ligand distribution on the facets of Au nanocrystals was conducted by atomic force microscopy infrared (AFM-IR) measurements, using gold-coated Si probes with a nominal diameter of ∼25 nm. AFM topography image of a single Au nanocrystal coated with p-NTP is shown in Figure 2A(I). Topography measurement was followed by AFM-IR mapping at 1336 cm^–1^, correlated to the N–O vibration (Figure 2A(II)).

**Figure 2:**
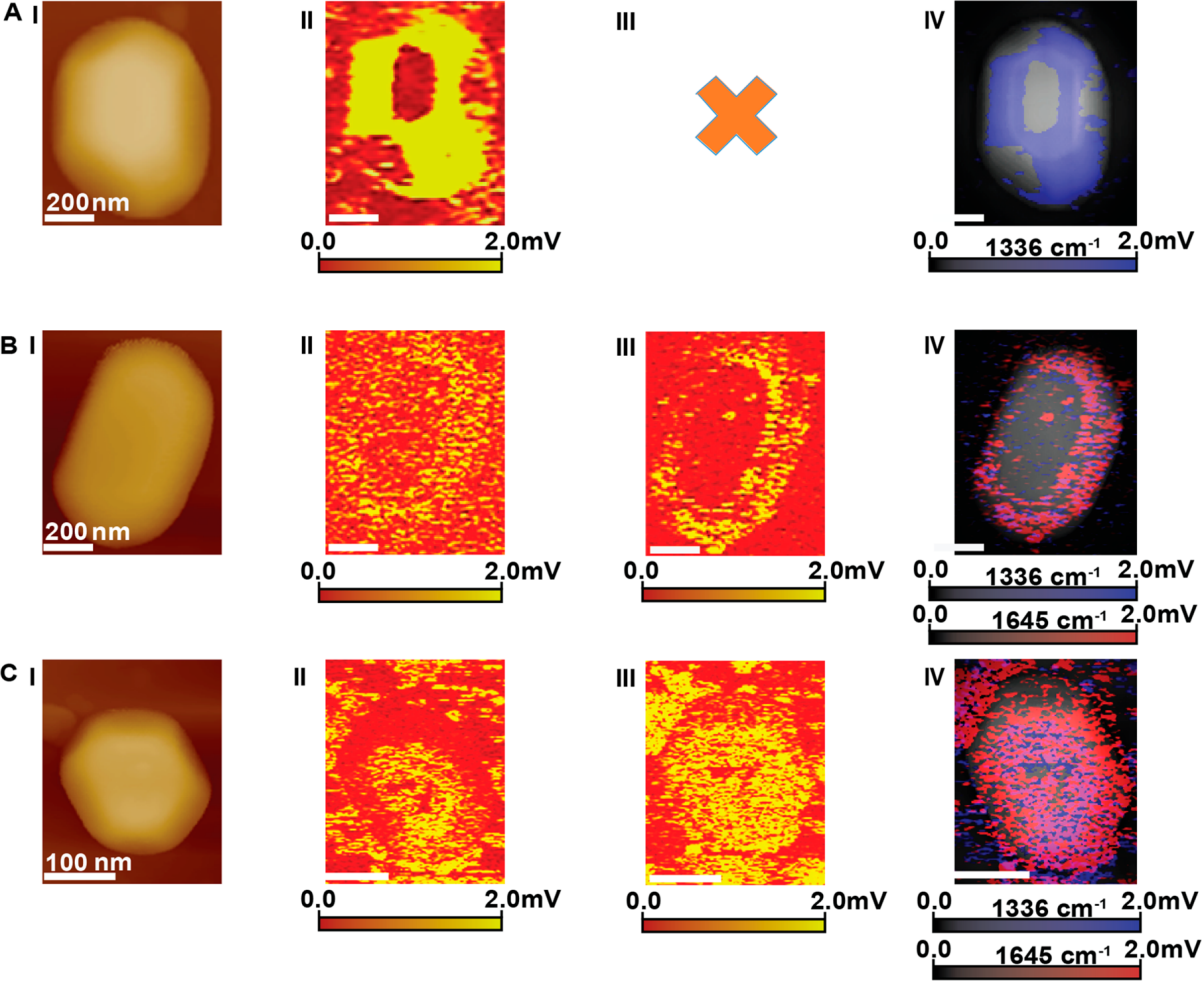
AFM and AFM-IR measurements of p-NTP on Au nanocrystals at room temperature (A(I)–A(IV)), after exposure to elevated temperature (100 °C, 1 atm N_2_, 10 h) (B(I)–B(IV)), and after exposure to reducing conditions (100 °C, 1 atm H_2_, 10 h) (C(I)–C(IV)). AFM (A(I), B(I), C(I)) and AFM-IR mapping at 1336 cm^–1^ (A(II), B(II), C(II)), correlated to the N–O symmetric stretch, and at 1645 cm^–1^ (A(III), B(III), C(III)), correlated to N–H bending, are presented. An overlay of the AFM-IR maps at 1336 and 1645 cm^–1^, colored in blue and red, respectively, and AFM topography images are shown in A(IV), B(IV), and C(IV).

AFM-IR map at 1336 cm^–1^ clearly shows an IR signal on the (001) facets, while a lower signal was detected on the (111) facets. The lower ligand adsorption affinity on the (111) facets was identified for various nanocrystals (Figure S1). AFM-IR mapping did not show any signal at 1645 cm^–1^, correlated to N–H vibration, indicative of the fact that NO_2_ reduction was not facilitated following p-NTP adsorption. IR spectra were locally acquired on different sites of the surface of the p-NTP coated Au nanocrystal and showed a single dominant peak at 1336 cm^–1^ (Figure S4). The peak amplitude decreased as the measurement location approached the top (111) facet, indicative of a lower surface density of p-NTPs on this facet.

Analysis of the IR signal amplitude on the nanocrystal revealed a similar IR amplitude on various (001) facets and interface sites, indicative of a comparable surface density of ligands on these sites (Figure S5). No IR signal was detected on the top (111) facet, while a low IR signal, which was 3-fold lower in its amplitude in comparison to the one detected on the (001) facets, was detected on the side (111) facets. Thus, variations in the surface density of ligands were detected on the side and top (111) facets and correlated to differences in the atomic roughness and uniformity of these facets. Homogeneous coverage of p-NTP was detected on amorphous Au nanoparticles, prepared by e-beam lithography, with no indication for selective adsorption (Figure S6).

The preferred adsorption of p-NTP on (001) facets can be rationalized based on DFT calculations that identified a higher stability of adsorbed thiolates on the more open (001) facets.^62^ Moreover, it was demonstrated that reconstruction of the Au(111) surface, by ejecting metal surface atoms to form thiolate–metal complexes, is essential for the formation of a chemisorbed monolayer with a Au–S-R bond.^63,64^ Reconstruction has not been experimentally observed on (100) surfaces following thiols’ adsorption due to the strong stability of thiols on these lattices.^65,66^ It is hypothesized that activation energy barriers prevented the reconstruction of the (111) facet at room temperature and therefore chemisorption of p-NTP on the nonreconstructed (111) facet has been kinetically mitigated.^67^

Au nanocrystals that were coated with p-NTP were exposed to elevated temperature (100 °C, 1 atm N_2_, 10 h) and characterized by AFM-IR measurements (Figure 2B(I)-(IV)). The signal in the AFM-IR images was lower than that measured prior to annealing (Figure S5). XPS measurements revealed that nitrogen concentration was reduced by 30% following annealing (Table S1), correlated to partial desorption of p-NTP.^65,68^ It should be noted that surface annealing also led to a noticeable nitro to amine reduction, and therefore the overall decrease in the AFM-IR signal at 1336 cm^–1^ (Figure 2B(II)) is also due to the appearance of a noticeable N–H signal at 1645 cm^–1^ (Figure 2B(III)). Integration of the two AFM-IR maps (Figure 2B(IV)) identifies that no dominant ligand diffusion toward the top (111) facet occurred following annealing. The deteriorated AFM-IR signal following annealing makes it challenging to clearly identify variations in the surface density of ligands on the (111) and (001) side facets. Therefore, the following discussion will mostly focus on variations in the ligands’ properties (i.e., distribution and reactivity) between the side facets and the top (111) facet.

Comparative analysis of the IR signal amplitude across the nanocrystal (Figure S5) showed a minor N–O vibration on the top (111) facet, which indicates that p-NTP diffusion toward the top (111) facet was initiated by annealing. The amplitude of the N–O vibration on the top (111) facet was ∼3 fold lower than the one measured at the interface and side facets. This result indicates that molecular diffusion was not a dominant process following annealing (Figure S5 and S7).

Exposure to elevated temperatures also led to partial reduction of the nitro groups, as detected by a dominant signal in the AFM-IR mapping at 1645 cm^–1^ (Figure 2B(III)). Nitro reduction can be associated with the presence of hydrogen atoms on the Au surface due to dissociation of S–H bond in the chemisorbed p-NTP.^69,70^ AFM-IR map of the N–H signal (Figure 2B(III)) was similar in its pattern to the AFM-IR map of the N–O signal (Figure 2B(II)), as shown in the AFM-IR overlay image (Figure 2B(IV)). Local enhancement in the N–H signal was detected at the interface of two facets (Figure S5) and correlated to higher density of surface defects at these sites.^47,49^

Single point IR measurements were conducted on the annealed p-NTP coated Au nanocrystal (Figure S4). In most measurements, the spectra included a dominant N–O vibration, while the N–H signal was detected on one spectrum. The lack of N–H signature in most single point IR measurements can be attributed to the fact that N–O reduction was typically facilitated at interfacet sites, on which the acquisition of IR spectrum is challenging due to deteriorated stability of the AFM tip on these sites.

Exposure of the sample to reducing conditions (100 °C, 1 atm of H_2_, 10 h) led to both p-NTP diffusion and nitro reduction (Figure 2C(I)–(IV)). AFM-IR map at 1336 cm^–1^ (Figure 2C(II)) probed the nitro-related signal on the top (111) facet, while no nitro-related signal was detected on the side facets. The AFM-IR map at 1645 cm^–1^ (Figure 2C(III)) revealed a homogeneous distribution of N–H vibration on the different facets. The AFM-IR maps therefore show that exposure to reducing conditions led to p-NTP diffusion toward the top (111) facet. However, nitro reduction occurred more effectively on the side facets (Figure 2C(IV)).

Changes in the chemical properties of p-NTP following exposure to reducing conditions were also identified in local IR nanospectroscopy measurements (Figure S4). The highest reactivity was detected on the side facets on which a dominant N–H vibration was probed, while a lower reactivity was detected on the top (111) facet. Comparative analysis of the IR signals across the nanocrystal showed a similar N–O and N–H signal amplitudes on the top (111) facet, while only N–H signals were detected on the side facets (Figure S5). N 1s XPS measurements (Figure S8 and Table S1) identified that ∼70% of the nitro groups were reduced following exposure to reducing conditions. These results validate the IR spectroscopy measurements that probed a noticeable N–O signature even after exposure to reducing conditions.

Analysis of the obtained results indicates that diffusion of p-NTP from the side facets toward the top (111) facet was facilitated under reducing conditions. Thus, it is hypothesized that ligand diffusion was accelerated by exothermic nitro reduction that provided sufficient thermal energy to enable the diffusion of surface ligands. The enhanced nitro reduction efficiency on the side facets can be correlated with higher affinity toward dissociative chemisorption of H_2_ on the (001) facet and on interface sites. It is expected that surface H atoms will desorb as H_2_ on the top (111) facet due to their weak interaction with this facet, thus locally lowering the nitro-to-amine reduction affinity.^71^

Integration of the experimental results for p-NTP adsorption, diffusion, and reduction on Au nanocrystals shows that there is a preferred adsorption of p-NTPs on the more open (001) facets and lower affinity toward adsorption on the side and top (111) facets. Elevated temperature led to partial reduction of the nitro groups, with an enhanced reduction yield at the facet interface. However, nitro reduction was not coupled to a dominant p-NTP diffusion to the top (111) facet. Exposure to reducing conditions further enhanced the nitro reduction and led to diffusion toward the top (111) facet. Deteriorated affinity toward nitro reduction was obtained on the top (111) facet even after exposure to reducing conditions. Nitro reduction was also studied on an extended Au film (Figure S9–S10), showing a reactivity pattern similar to that of the side facets of Au nanocrystals. This similarity was correlated to high density of defects on the Au film that facilitated hydrogen dissociation and nitro reduction.

While p-NTP is chemically coordinated to the Au surface, nitro-naphthalene is stabilized by VdW interactions.^61^ Thus, comparative analysis of the properties of p-NTP versus nitronaphthalene will uncover the impact of ligand–metal interactions on the adsorption, diffusion, and reactivity of surface ligands. AFM-IR imaging of the N–O vibration following adsorption of nitronaphthalene on the Au nanocrystal showed a preferred adsorption on the side facets, with a lower vibrational signal on the top (111) facet (Figure 3A(I), (II)). No signal was detected in the AFM-IR map at 1645 cm^–1^, which indicates that N–O reduction was not induced following adsorption (Figure 3A(III)). Single point IR measurements (Figure S11) showed a dominant vibrational signature at 1340 cm^–1^, correlated to the N–O vibration, and similar vibrational signatures were probed on the different side facets (Figure S12). A single spectral measurement showed a minor peak at 1595 cm^–1^, which can indicate a partially reduced nitroso specious.^72^

**Figure 3:**
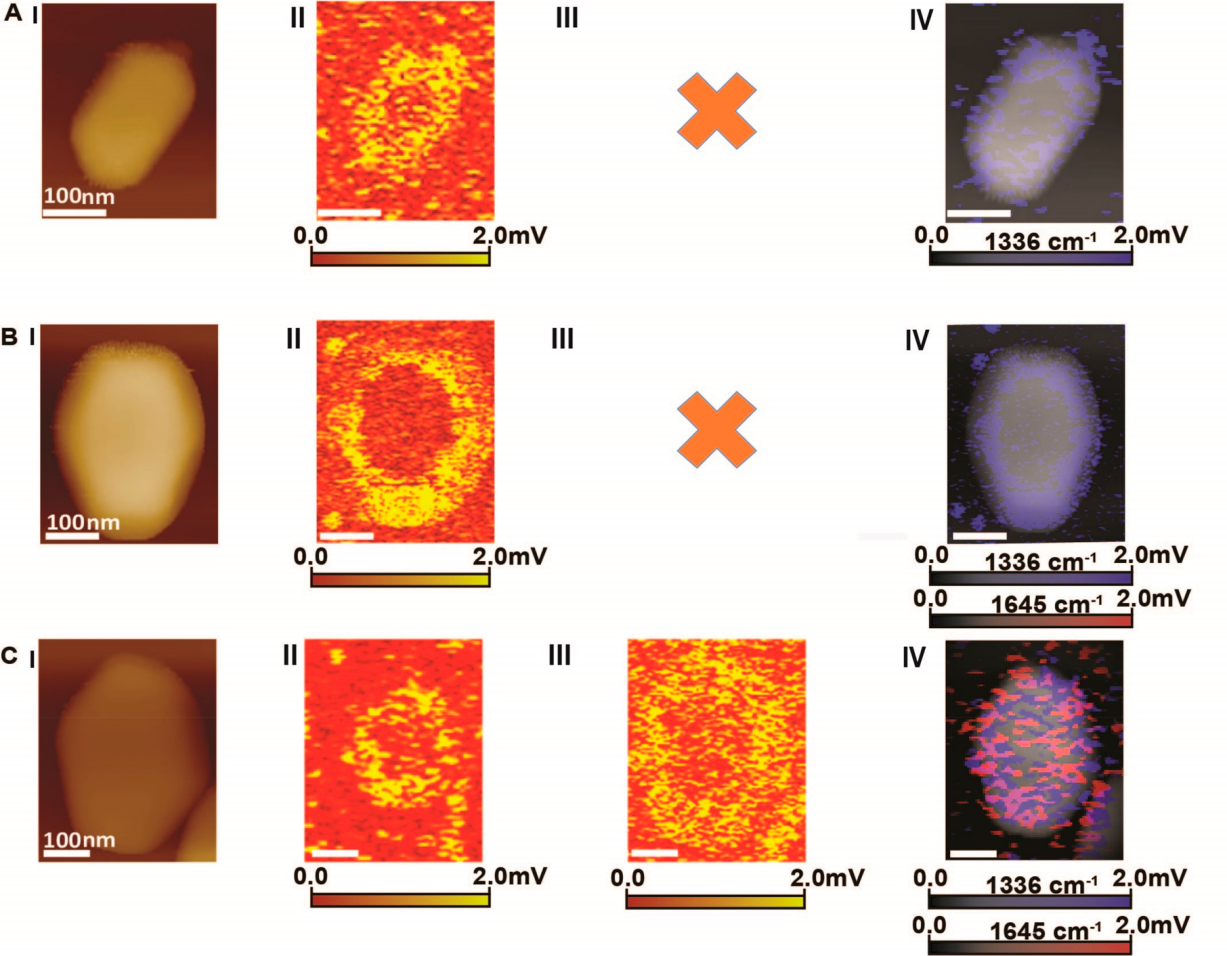
AFM and AFM-IR measurements of nitronaphthalene on Au nanocrystals (A(I)–A(IV)), after exposure to elevated temperature (100 °C, 1 atm N_2_, 10 h) (B(I)–B(IV)) and after exposure to reducing conditions (100 °C, 1 atm H_2_, 10 h) (C(I)–C(IV)). AFM (A(I), B(I), C(I)) and AFM-IR mapping at 1336 cm^–1^ (A(II), B(II), C(II)), correlated to the N–O symmetric stretch, and at 1645 cm^–1^ (A(III), B(III), C(III)), correlated to N–H bending, are presented. An overlay of the AFM-IR maps at 1336 and 1645 cm^–1^, colored in blue and red, respectively, and AFM topography image are shown in A(IV), B(IV) and C(IV).

No major changes in ligand distribution were detected after exposure of the sample to elevated temperature (100 °C, 1 atm N_2_, 10 h), with almost no vibrational signal on the top (111) facet in the AFM-IR mapping at 1336 cm^–1^ (Figure 3B(II)). Differences were probed in ligand distribution on the side facets, with an alternating adsorption mode that favors adsorption on the (001) facet and was also detected at room temperature for p-NTP on Au nanocrystals (Figure 2A(II)). Enhanced N–O signal was detected at the interface of the two facets, which indicates that ligand diffusion toward the interface has occurred. AFM-IR mapping at 1645 cm^–1^ did not show any signal, demonstrating that N–H formation was not favorable under these conditions (Figure 3B(III)), as also identified in single point IR measurements (Figure S11).

Thus, unlike p-NTP, nitro reduction was not detected for nitronaphthalene following exposure to an elevated temperature. This difference in the reactivity pattern of the two ligands can imply that the source for p-NTP reduction was atomic hydrogen that was present on the surface following S–H dissociation. Moreover, analysis of the IR signal amplitude showed that, unlike for p-NTP, annealing did not lead to thermal desorption of nitronaphthalene or to its diffusion toward the top (111) facet (Figure S12). These results demonstrate the lower diffusivity of nitronaphthalene and the crucial impact of surface–adsorbate VdW interactions in ligand stabilization.

Diffusion and nitro reduction of nitronaphthalene were detected after exposure to reducing conditions (100 °C, 1 atm H_2_, 10 h), as identified in AFM-IR mapping (Figure 3C). The AFM-IR map of the N–O vibration (Figure 3C(II)) shows the diffusion of nitronaphthalene toward the top (111) facet. The AFM-IR map of N–H vibration (Figure 3C(III)) identified that molecules that were adsorbed at the interface and at the side facets were fully reduced after exposure to reducing conditions. However, no reduced molecules were detected on the central part of the top (111) facet. A similar reactivity trend was identified by single point IR measurements that showed noticeable nitro to amine reduction (Figure S11). Comparative analysis of the IR signals (Figure S12) showed that after exposure to reducing conditions the surface density of molecules on the top (111) facet was 3–4 times lower than that of the side facets, thus indicating that molecular diffusion to the top (111) facet was initiated.

It can be therefore concluded that both p-NTP and nitronaphthalene show a lower affinity toward adsorption on the top (111) facets. The improved adsorption affinity on the (001) facet was correlated to the less-rigid and more open atomic structure of this facet.^73^ Exposure to elevated temperature did not provide sufficient energy to induce diffusion of the two ligands toward the top (111) facet. However, differences were identified in the reducibility of the nitro groups of the two ligands. The presence of hydrogen atoms on the Au surface, following the surface anchoring of p-NTP, enabled nitro reduction during surface annealing, while no reduction was detected for nitronaphthalene.

Exposure to a reducing environment led to nitro reduction and ligand diffusion of p-NTP and nitronaphthalene. For both ligands, diffusion toward the top (111) facet and deteriorated reactivity on this facet were detected. Nitro reduction in p-NTP was identified on all facets, while the reduced species of nitronaphthalene was not detected on the top (111) facet. The enhanced affinity toward nitro reduction in p-NTP was also obtained in ensemble-based IR measurements (Figure S13).

Higher diffusivity was probed for p-NTP, and after exposure to reducing conditions the top (111) facet was coated with a 1:1 ratio of nitro- and amine-functionalized thiol ligands, and a homogeneous coverage of the amine-functionalized thiol was detected on various facets on the nanocrystal (Figure S5). Lower diffusivity was detected for nitronaphthalene, and a 3:1 ratio in IR signal amplitude was detected when comparing the IR signature of amine-naphthalene on the side and top (111) facets after exposure to reducing conditions, with a close to noise level nitronaphthalene signature on the central part of the top (111) facet (Figure S12).

The enhanced diffusivity of p-NTP is attributed to its strong interaction with the Au surface that can lead to an adatom adsorption mode which facilitates ligand diffusion toward the top (111) facet.^32,74^ The diffusion of nitronaphthalene involves cleavage of multiple VdW interactions with the underlying surface atoms, and therefore, lower diffusion rates were expected for this ligand. While no reactivity was detected for nitronaphthalene on the top (111) facet, a noticeable reactivity was detected for p-NTP on this facet. This variation is attributed to diffusion of both nitro- and aminothiophenol from the side facets.

The differences in ligand–metal interactions of p-NTP and nitronaphthalene not only directed their diffusivity and reactivity on Au nanocrystals but also led to major differences in ligand diffusion beyond the boundaries of Au nanocrystals. AFM-IR images showed noticeable N–O and N–H signals on the silicon surface following exposure of p-NTP coated Au nanocrystals to reducing environment (Figure 4A–C), and AFM measurements detected the formation of Au nanoparticles (Figure 4D). The detection of these nanoparticles is indicative of thiol-Au complex diffusion that led to the formation of thiol-coated Au nanoparticles. Diffusion of ligands or Au atoms toward the Si surface was not detected for nitronaphthalene coated nanocrystals (Figure S14), in which both N–O and N–H signals were confined to the Au nanocrystals, even after exposure to reducing conditions.

**Figure 4:**
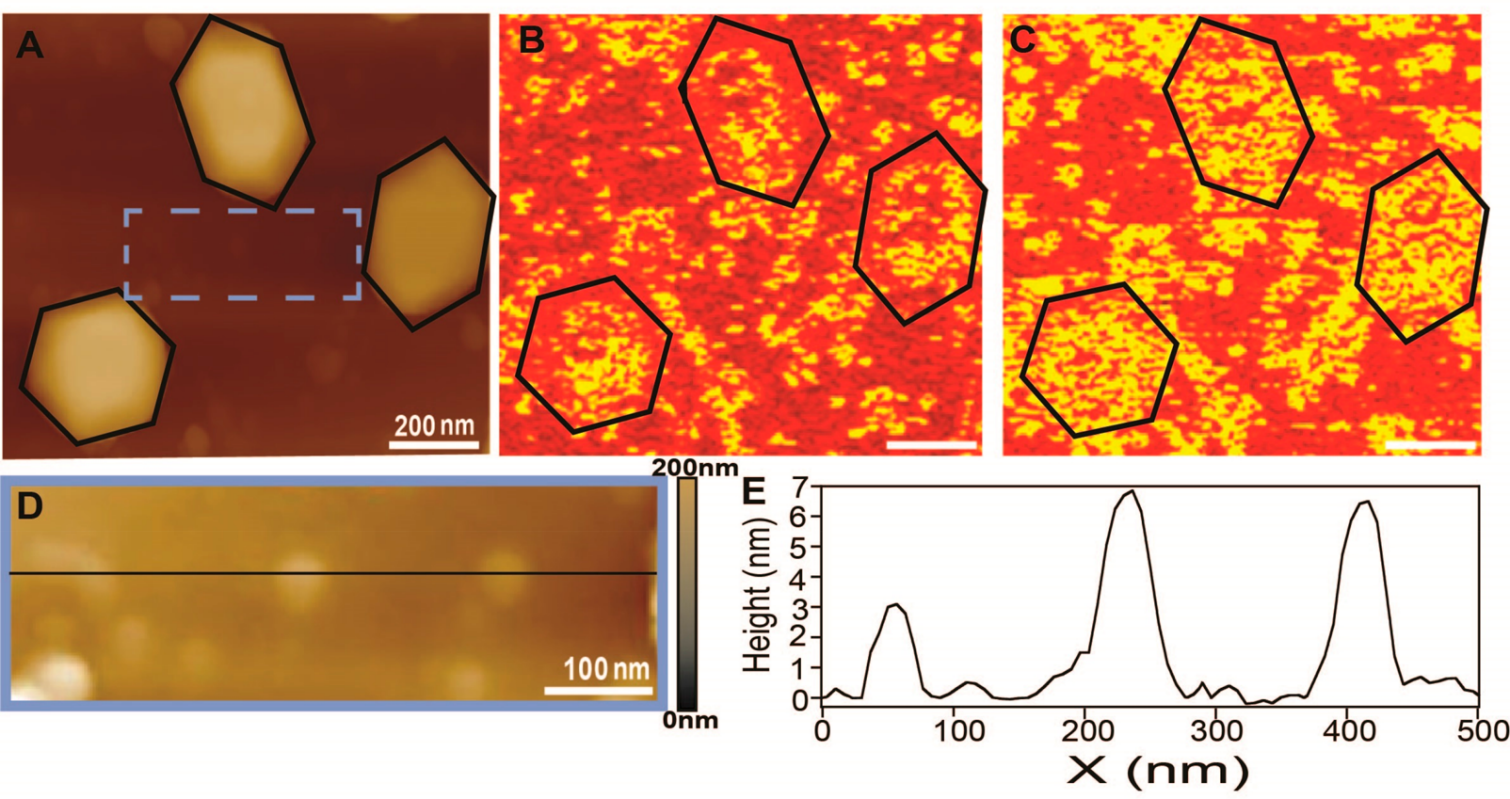
AFM (A) and AFM-IR mapping at 1336 cm^–1^ (B) and 1645 cm^–1^ (C) of Au nanocrystals on Si that were coated with p-NTP and exposed to reducing conditions (1 atm H_2_, 100 °C, 10 h). (D) Zoom-in of the area highlighted by a blue rectangular in (A). (E) Height profile of the line marked in (D).

It is hypothesized that the strong interaction between p-NTP and Au surface atoms led to the formation of the p-NTP Au-atom complex,^32,75−77^ which enabled p-NTP diffusion to the silicon surface. A similar process was recently demonstrated for N-heterocyclic carbene molecules on a Au film in which surface diffusion of Au-NHC complex was identified.^78^ The VdW interactions between nitronaphthalene and several Au surface atoms circumvented its diffusion beyond the Au nanocrystal boundaries.^61^

To conclude, in this work, we identified the crucial impact of atomic facets and metal–ligand interactions on the adsorption, diffusion, and reactivity of nitro-functionalized ligands on Au nanocrystals. Preferred adsorption on the (001) facet was probed for both p-NTP and nitronaphthalene and correlated to stronger interactions with the more open (001) facet. Deteriorated nitro reduction efficiency was probed for both ligands on the top (111) facet and was attributed to a lower hydrogen dissociation affinity on this facet. Nitro reduction in p-NTP was observed after exposure to an elevated temperature and was associated with the presence of surface H atoms that were present due to S–H dissociation following thiol adsorption. In addition to their enhanced reactivity, p-NTP molecules also showed higher diffusivity and fully coated the Au nanocrystals and were also detected on the Si surface after exposure to reducing conditions. The higher diffusivity was associated with the strong interaction of p-NTP and the Au surface, which led to the formation of the p-NTP gold adatom complex that is characterized with high surface mobility. It is therefore identified that the atomic order in each facet has a crucial impact on the ligand’s adsorption and reactivity, while ligand–metal interactions play a dominant role in determining the ligand’s diffusivity. Thus, both the atomic arrangement of the facet and ligand–metal interactions should be considered in the rational design of nanocrystal growth and surface manipulation.^79−81^

## Supporting Information Available

Additional AFM-IR images, ATR spectra of ligands, IR nanospectroscopy measurements on single particles, IR signal analysis of single nanoparticles, XPS and PM-IRRAS measurements (PDF)
