# Supplementary material for: Nanoimaging of Facet-Dependent Adsorption, Diffusion, and Reactivity of Surface Ligands on Au Nanocrystals
Source: Nano Lett. 2023 Jun 16;23(12):5437–44. doi: 10.1021/acs.nanolett.3c00250 (PMC10311598; doi:10.1021/acs.nanolett.3c00250)
Supplement: Supplementary file 1 — nl3c00250_si_001.pdf [file nl3c00250_si_001.pdf]

Supporting Information

Nanoimaging of Facet-Dependent Adsorption, Diffusion and Reactivity of Surface Ligands on Au Nanocrystals

Lihi Rikanati, Hadar Shema, Tzipora Ben-Tzvi, and Elad Gross*

Institute of Chemistry and The Center for Nanoscience and Nanotechnology, The Hebrew University, Jerusalem 91904, Israel

* Corresponding author email address: elad.gross@mail.huji.ac.il

Experimental details

15 nm thick Au film was evaporated on a Si wafer at room temperature, followed by annealing (750 °C, 1 atm N_2, 6 hr). The samples were immersed in a Piranha solution (90% concentrated H_2SO_4 and 10% concentrated (i.e., 35%) H_2O_2) followed by rinsing with triple-distilled water. P-Nitrothiophenol (*p*-NTP), and nitronaphthalene were purchased from Alfa-Aesar and Merck, respectively, and used without additional purification. The sample was immersed in a solution of *p*-NTP or nitronaphthalene (5 mM in ethanol, rt, 24 hr). After immersion, the sample was removed from the solution and rinsed with ethanol.

AFM-IR measurements were performed at tapping mode using a nanoIR-3 setup (Anasys, Bruker) equipped with a Bruker Hyperspectral QCL laser source (790–1950 cm^{-1}), gold-coated Si probes with a nominal diameter of ~25 nm, resonance frequencies of 75±15 kHz, and spring constants of 1-7 N m^{-1}. Averaged spectral acquisition time was 5 sec per spectrum with a spectral resolution of 2 cm^{-1}. All nano-IR measurements were conducted at room temperature under nitrogen atmosphere.

Table S1: N1s XPS data analysis of p-NTP on Au nanocrystals

	**Nitrogen atomic percentage (%)**	**NO:NH peaks area ratio**
**As-deposited**	1.9	1.4
**After annealing**	1.4	1.1
**After reduction**	1.3	0.7

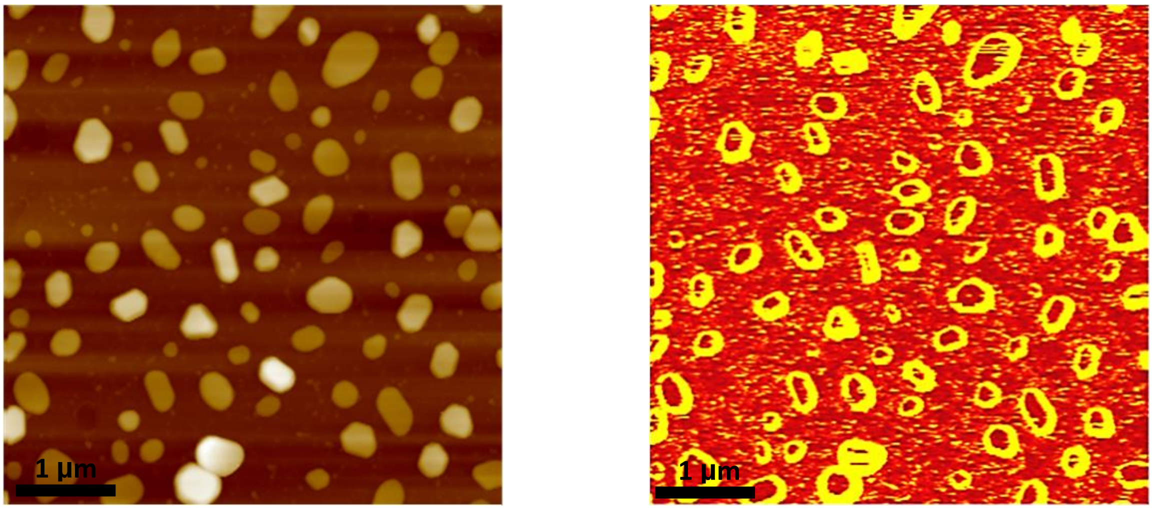

Figure S1. AFM image of Au nanocrystals prepared by evaporating 15 nm thick Au film on Si (110) wafer followed by annealing (750 °C, 1 atm N_2, 6 hr), coated with p-NTP (left image). AFM-IR mapping at 1336 cm^{-1}, which is correlated to N-O vibration (right image).

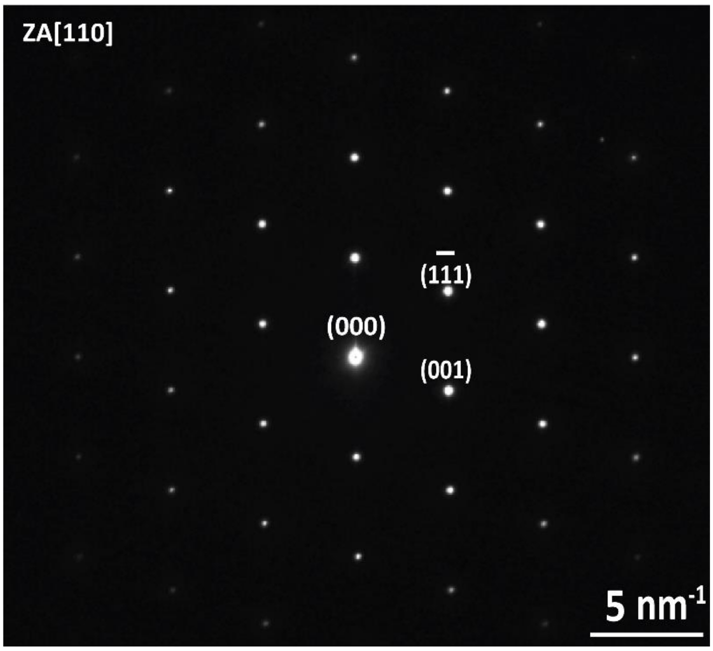

Figure S2. Diffraction pattern of the extracted lamella of a single Au nanocrystal with indexing facets.

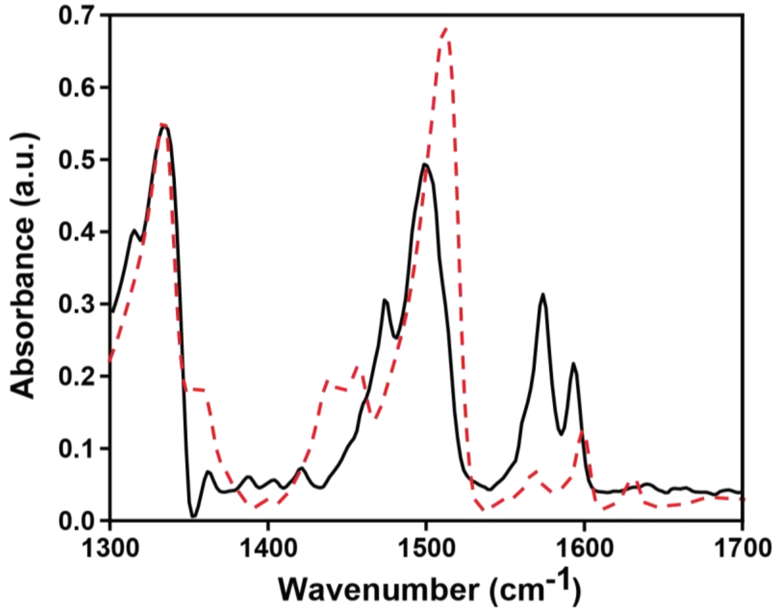

Figure S3. ATR-IR spectra of p-NTP (black-colored spectrum) and nitronaphthalene (red-colored spectrum).

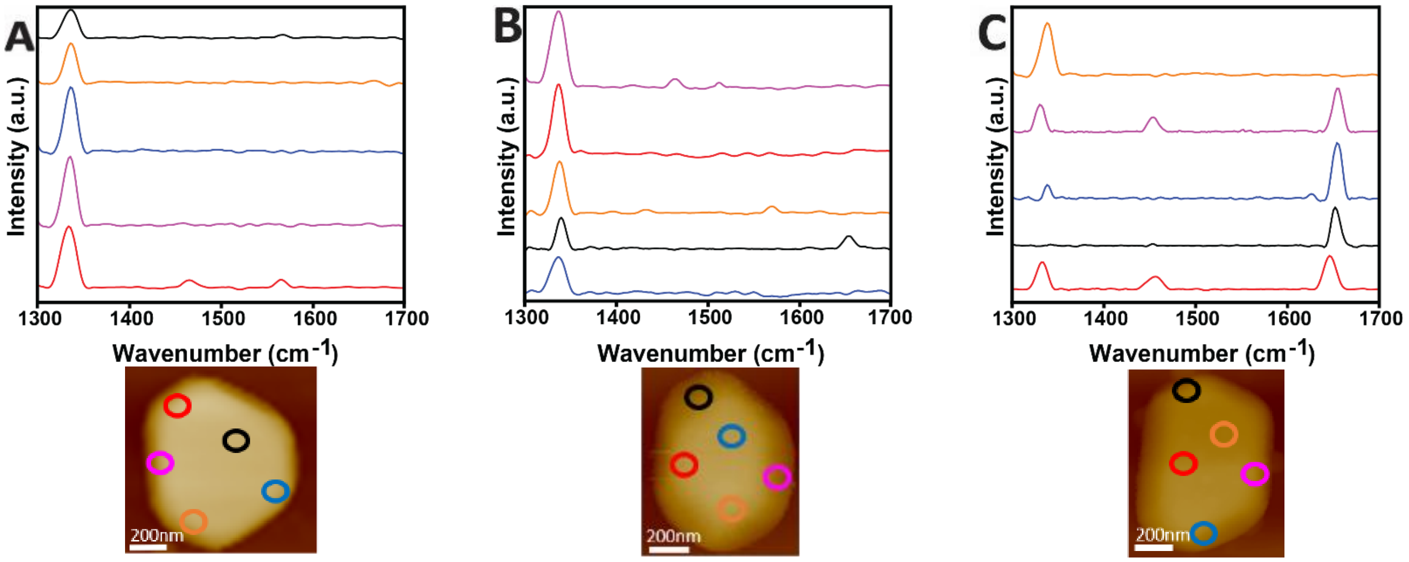

Figure S4. IR spectra were acquired on different sites on the surface of Au nanocrystal following p-NTP adsorption (A), after annealing to 100 °C (B), and after exposure to reducing conditions (100 °C, 1 atm H_2, 10 hr) (C). Color coding marks the position in which each spectrum was acquired.

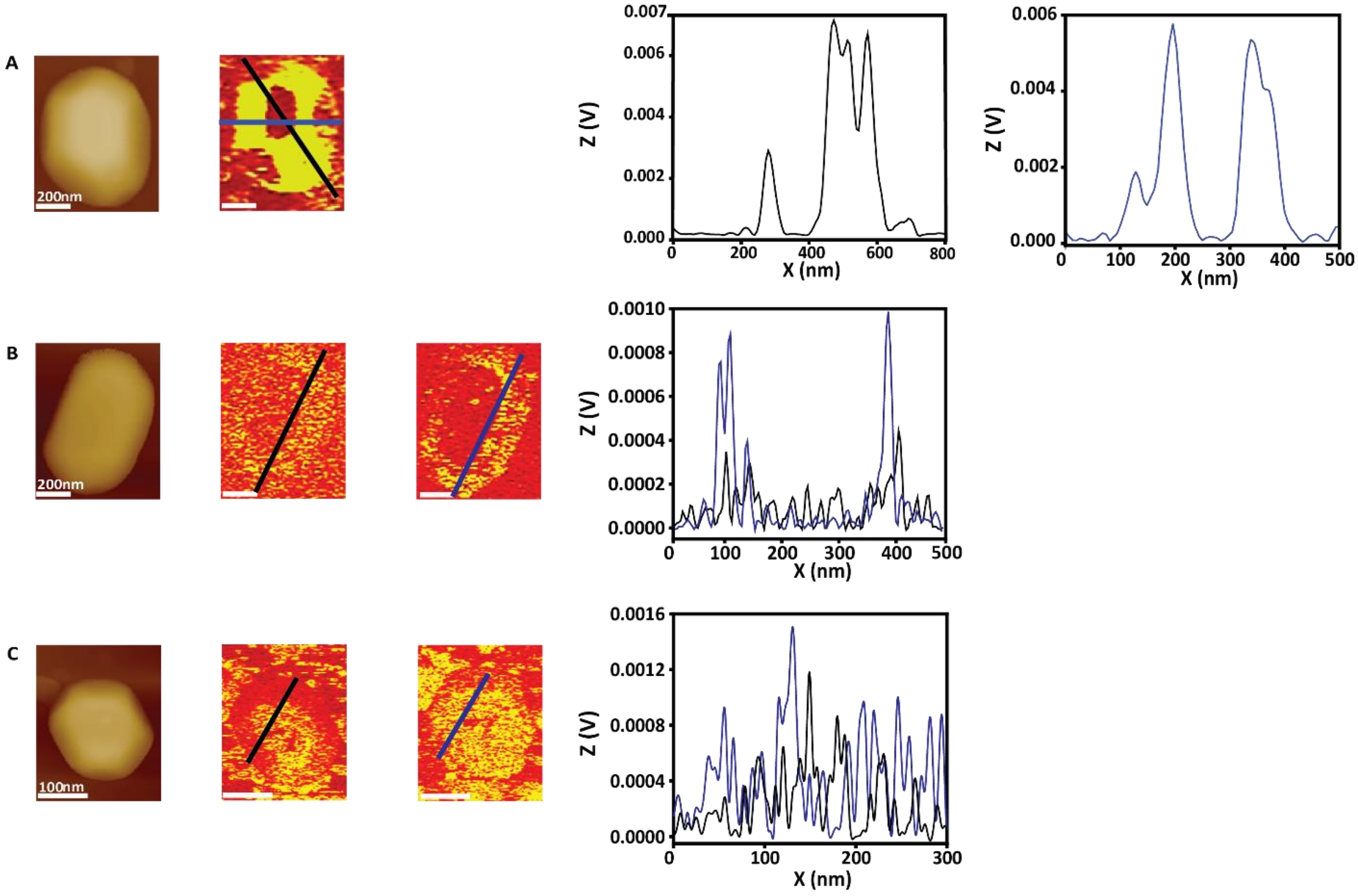

Figure S5. IR signal amplitude analysis on p-NTP coated nanocrystals (A), after annealing to 100 °C (B), and after exposure to reducing conditions (100 °C, 1 atm H_2, 10 hr) (C). IR signal analysis was performed along the color-coded lines marked in the AFM-IR maps.

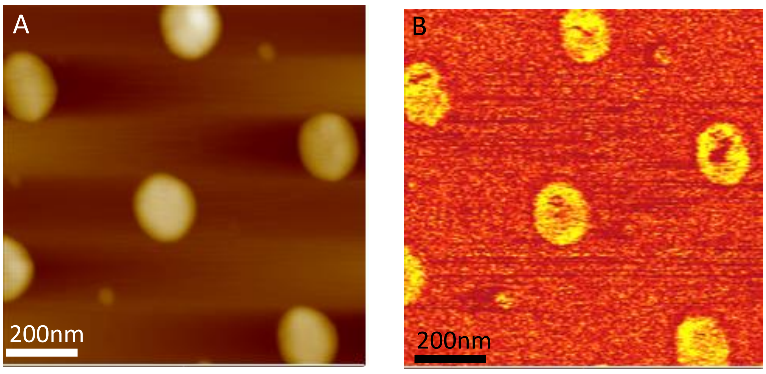

Figure S6. **A**. AFM topography measurement of Au nanoparticles that were prepared by e-beam lithography and coated with p-NTP. **B**. AFM-IR mapping at 1336 cm^{-1}, correlated to the symmetric stretch of N-O vibration.

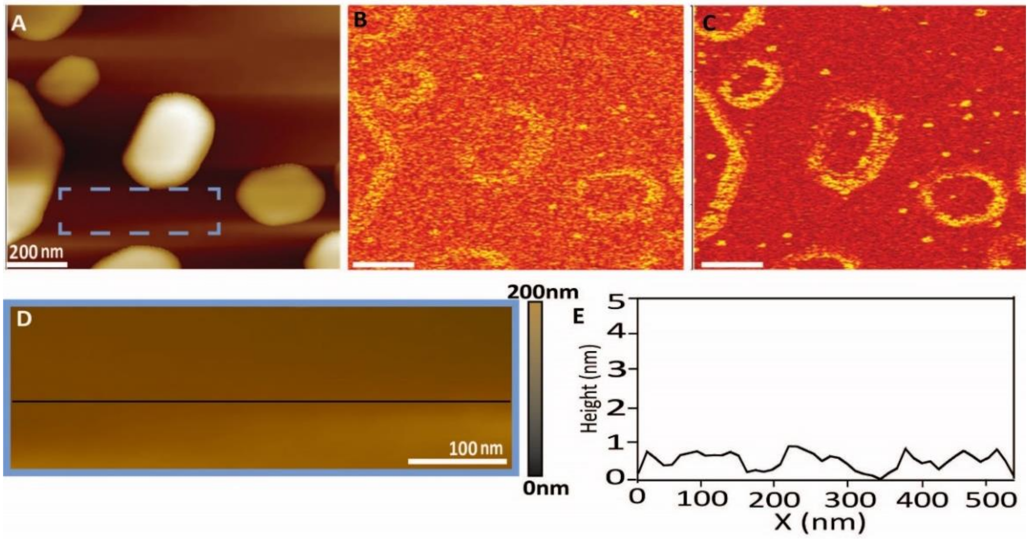

Figure S7. AFM topography (A) and AFM-IR maps at 1336 (B) and 1645 cm^{-1} (C) of Au nanocrystals on Si that were coated with p-NTP and exposed to elevated temperature (100 °C, 10 hr). D. Zoom-in of the area highlighted by blue rectangular in A. E. height profile of the line marked in D.

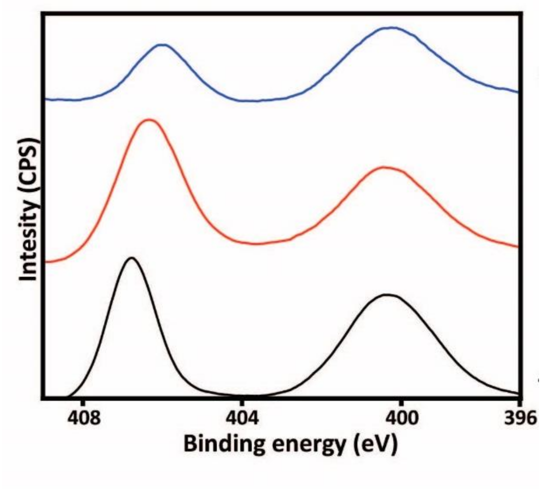

Figure S8. N1s XPS measurements of p-NTP coated Au nanocrystals at room temperature (black-colored), after annealing to 100 °C (red-colored), and after exposure to reducing conditions (100 °C, 1 atm H_2, 10 hr) (blue-colored).

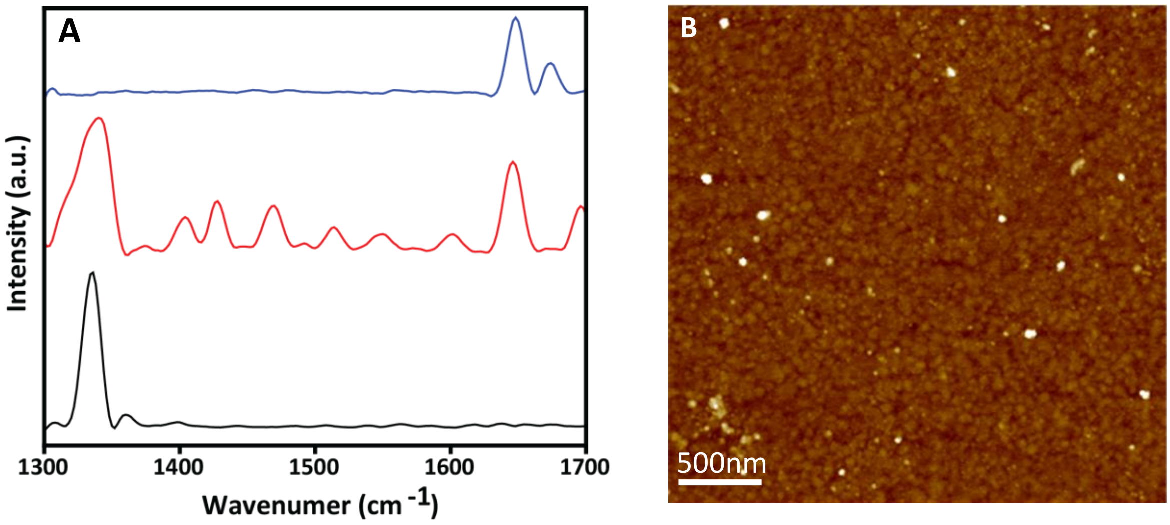

Figure S9. A. IR spectra were acquired on extended Au film following p-NTP adsorption (black-colored), after annealing to 100 °C (red-colored), and after exposure to reducing conditions (100 °C, 1 atm H_2, 10 hr) (blue-colored). **B.** AFM topography of Au film.

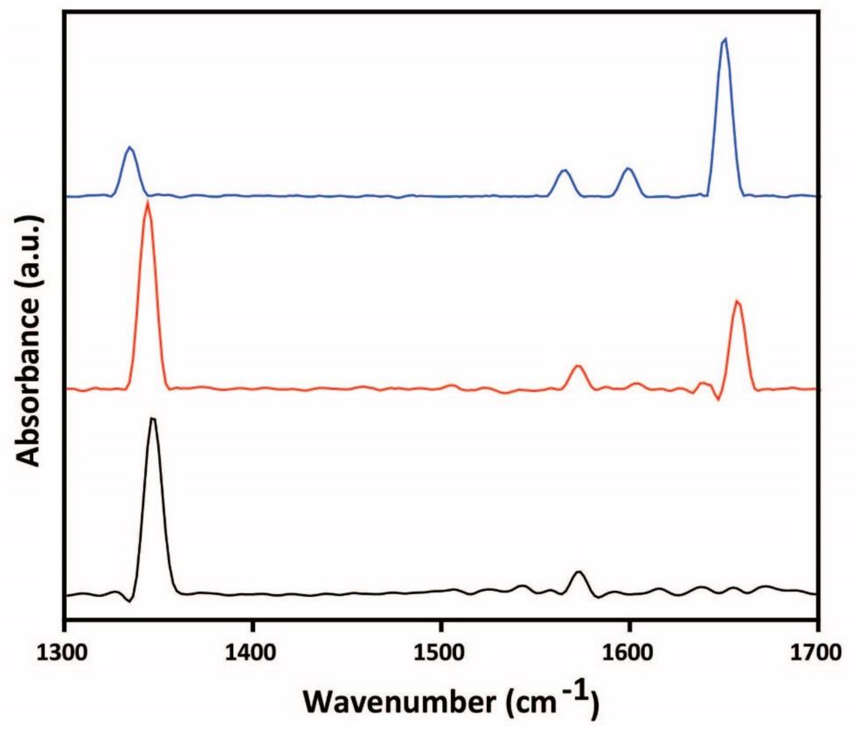

Figure S10. PM-IRRAS measurements were acquired on extended Au film following p-NTP adsorption (black-colored), after annealing to 100 °C (red-colored), and after exposure to reducing conditions (100 °C, 1 atm H_2, 10 hr) (blue-colored).

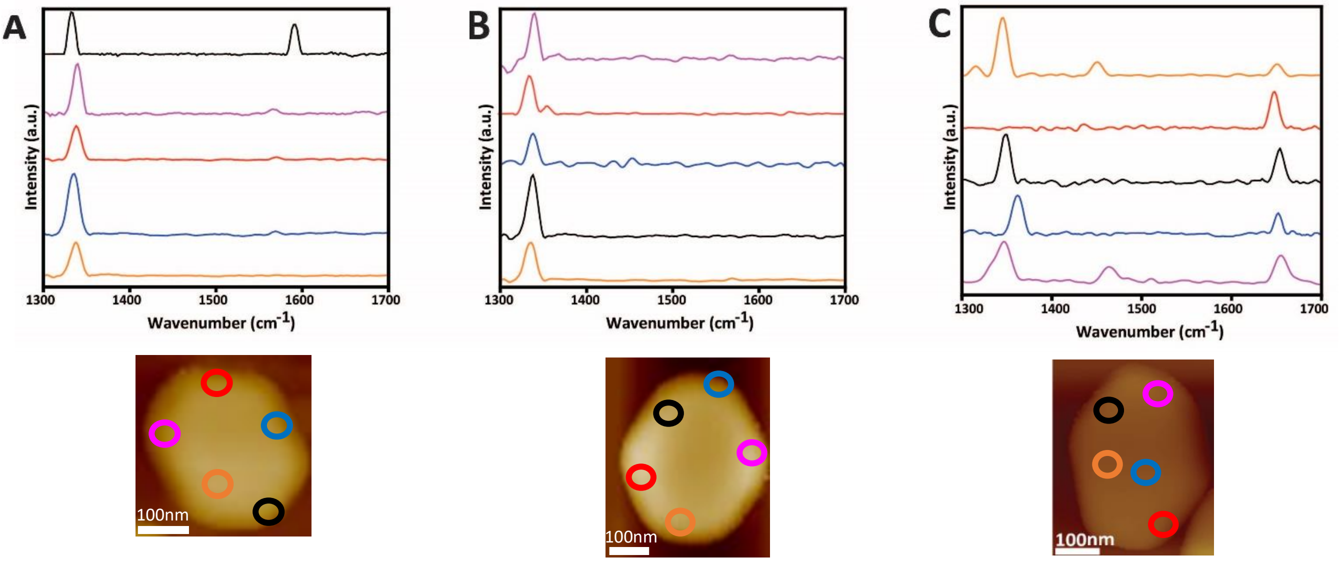

Figure S11. IR spectra were acquired on different sites on the surface of Au nanocrystal following nitronaphthalene adsorption (A), after annealing to 100 °C (B), and after exposure to reducing conditions (100 °C, 1 atm H_2, 10 hr) (C). Color coding marks the position in which each spectrum was acquired.

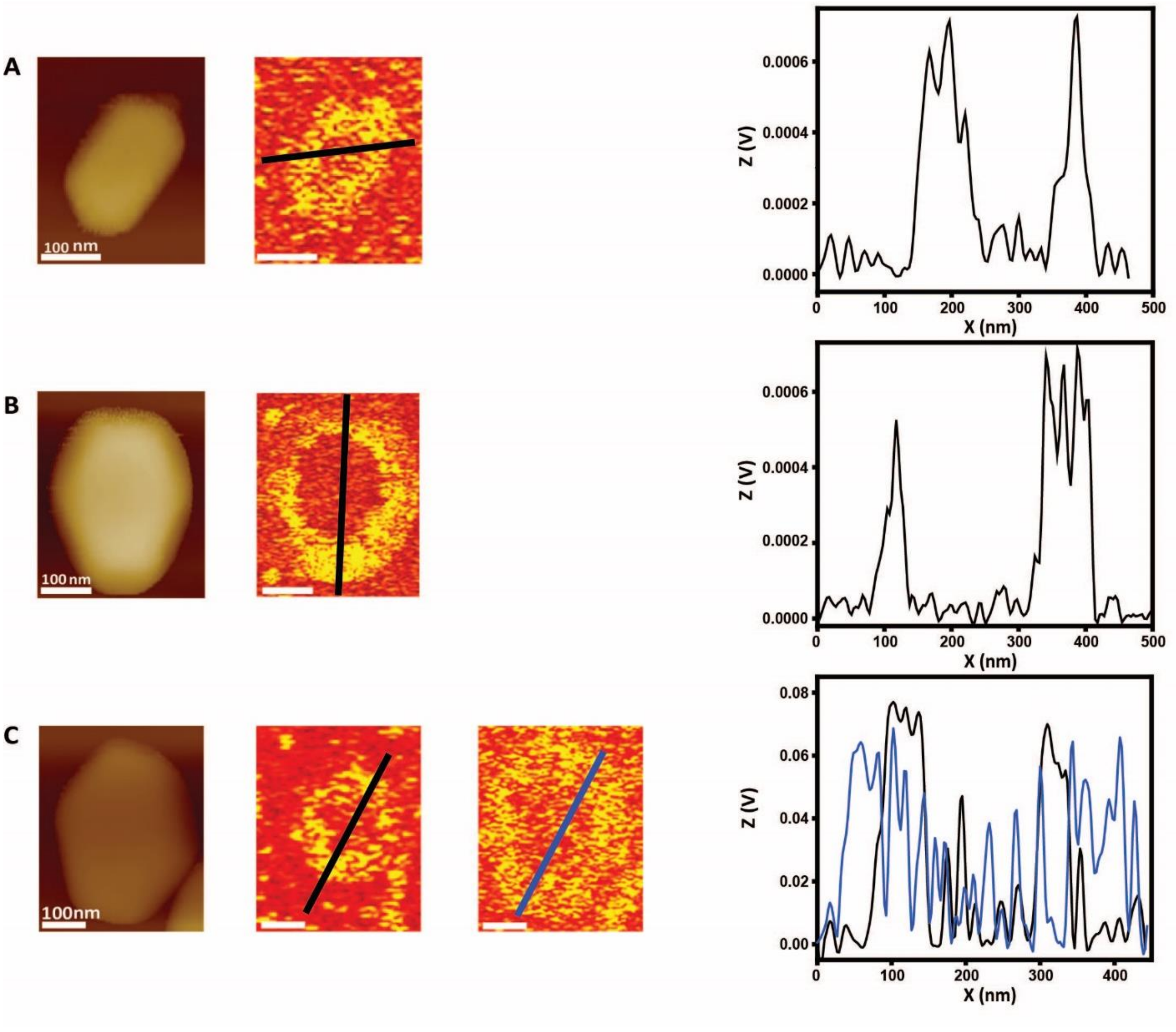

Figure S12. IR signal amplitude analysis of nitronaphthalene-coated nanocrystals (A), after annealing to 100 °C (B), and after exposure to reducing conditions (100 °C, 1 atm H_2, 10 hr) (C). IR signal analysis was performed along the color-coded lines marked in the AFM-IR maps.

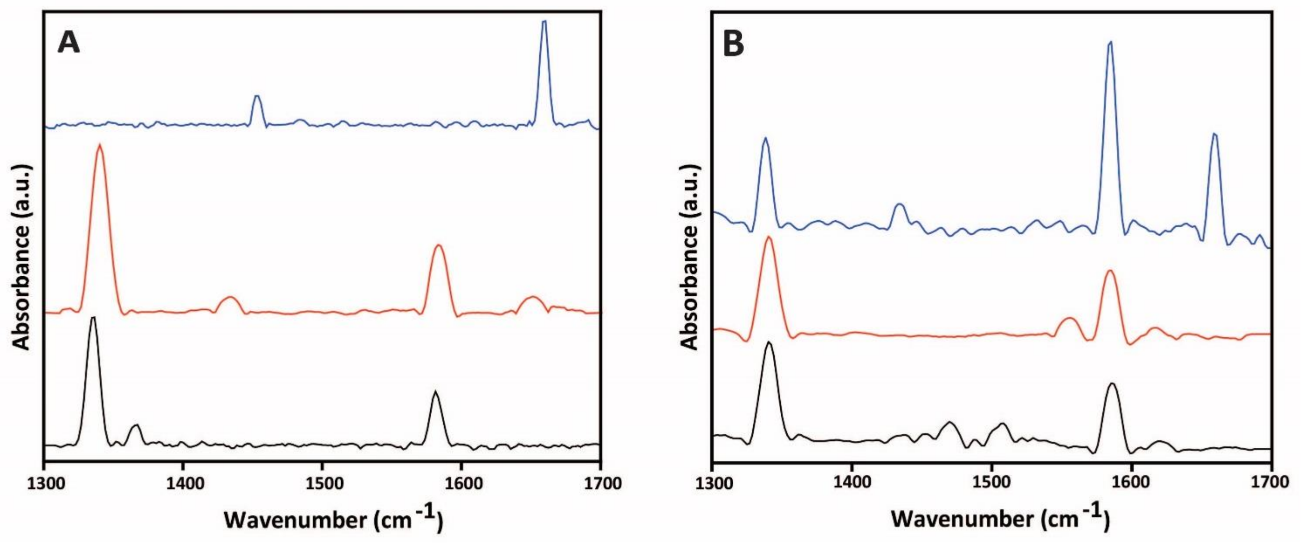

Figure S13. PM-IRRAS measurements were acquired on Au nanocrystals after p-NTP (A) and nitronaphthalene (B) adsorption (black-colored), annealing to 100 °C (red-colored), and exposure to reducing conditions (100 °C, 1 atm H_2, 10 hr) (blue-colored).

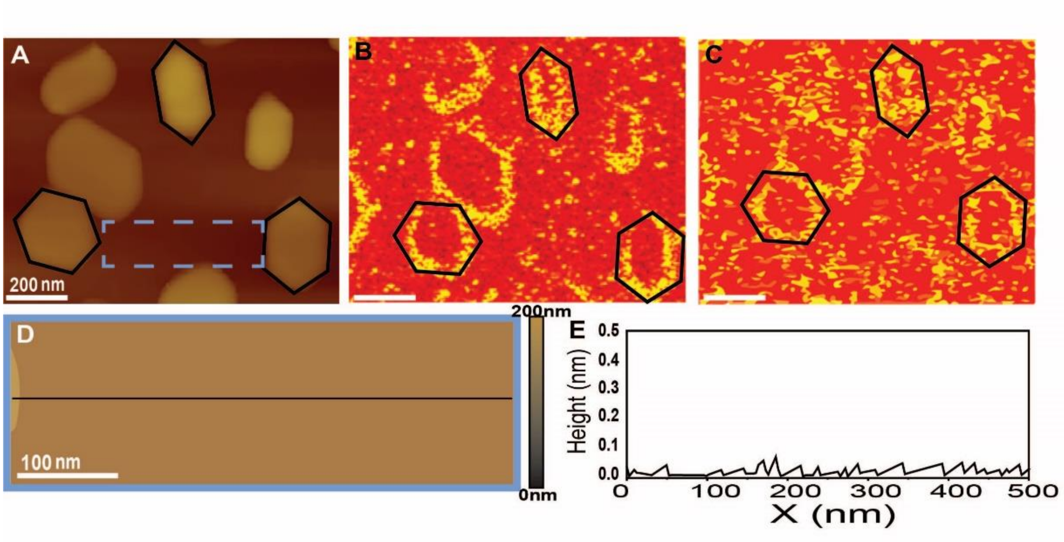

Figure S14. AFM topography (A) and AFM-IR maps at 1336 cm^{-1} (B) and 1645 cm^{-1} (C) of Au nanocrystals on Si that were coated with nitronaphtalane and exposed to reducing conditions (1 atm H_2, 100 °C, 10 hr). D. Zoom-in of the area highlighted by blue rectangular in A. E. height profile of the line marked in D.
